# Circadian Oscillation of Leukocyte Subpopulations and Inflammatory Cytokines over a 24-H Period in Horses

**DOI:** 10.3390/vetsci12040386

**Published:** 2025-04-20

**Authors:** Francesca Aragona, Maria Rizzo, Elisabetta Giudice, Francesco Fazio, Antonino Costa, Beatrice Di Bella, Salvatore De Caro, Francesca Arfuso, Marilena Briglia, Giuseppe Piccione, Claudia Giannetto

**Affiliations:** 1 Department of Veterinary Science, University of Messina, Via Giovanni Palatucci, 98168 Messina, Italy; fraragona@unime.it (F.A.); egiudice@unime.it (E.G.); ffazio@unime.it (F.F.); antonino.costa1@studenti.unime.it (A.C.); farfuso@unime.it (F.A.); gpiccione@unime.it (G.P.); clgiannetto@unime.it (C.G.); 2 Department of Engineering, University of Messina, C/da di Dio (S. Agata), 98166 Messina, Italy; beatrice.dibella@unime.it (B.D.B.); salvatore.decaro@unime.it (S.D.C.); 3 Department of Medicine and Surgery “Kore”, University of Enna, 94100 Enna, Italy; marilena.briglia@unikore.it

**Keywords:** horse, circadian rhythms, immune system, leukocyte subpopulation, inflammatory response, CD4-CD8, cytokine, interleukins

## Abstract

The objective of the present study was to investigate the influence of daily rhythms on the immune and inflammatory systems in horses, considering white blood cell count (WBCs), leukocyte subpopulations (neutrophils, basophils, eosinophils, lymphocytes, and monocytes), CD4+, and CD8+ lymphocyte populations, interleukin-1β (IL-1β), interleukin-6 (IL-6), and tumor necrosis factor-α (TNF-α). Ten Italian Saddle horses (7–12 years old, body weight 480 ± 30 kg) underwent blood sampling every 4 h over a 24-h period. The COSINOR method was used to identify rhythms and their parameters. A one-way analysis of variance (ANOVA) was applied to identify the differences in acrophase and robustness, and a multiple correlation analysis model (Pearson) was used to evaluate the relationships among the investigated parameters. WBCs, leukocyte subpopulations, CD4+, CD8+, IL-1β, IL-6, and TNFα exhibited daily rhythmicity. In particular, white WBCs, lymphocytes, IL-1β, and IL-6 reached their acrophases during the dark phase, while neuthrophils, CD4+, CD8+, and TNFα showed a diurnal acrophase. One-way ANOVA showed a statistical difference in the acrophase among the investigated parameters (*p* < 0.0001). The Pearson correlation matrix showed positive and negative relationships among the parameters. Circadian rhythms should be taken into consideration with the daily fluctuations in immune and inflammatory biomarkers to develop good management practices and improve welfare in horses.

## Simple Summary

The temporal organization of biological processes in living organisms follows a specific rhythmicity governed by an endogenous circadian pacemaker, which is an evolutionarily adapted survival mechanism. In recent years, the connection between 24-h biological rhythms, immune pathways, and inflammatory responses has been demonstrated. The present study investigated the daily rhythmicity of white blood cell (WBC) counts, leukocyte subpopulations, CD4+ and CD8+ populations, interleukin-1β (IL-1β), interleukin-6 (IL-6), and tumor necrosis factor-α (TNF-α) in horses. Statistical analysis showed that WBCs and IL-1β exhibited a nocturnal acrophase, IL-6 and lymphocytes peaked during the early morning hours, and neutrophils, CD4+, CD8+, and TNF-α showed a diurnal acrophase. All parameters showed a robustness of rhythm higher than 70%. This finding is the first step toward understanding how the daily rhythm physiology of horses is closely related to their immunological and inflammatory responses. Studying the rhythms of these parameters can provide a possible window of risk for infections and inflammation to which horses may be susceptible. This is essential for formulating effective management or training regimens for equine athletes, as well as for disease prevention.

## 1. Introduction

Daily activities in domestic horses, including management practices, training, transportation, and competitions, can impact their normal physiological functions and consequently disrupt homeostasis through a complex network of reactions associated with the inflammatory response [1,2,3,4,5,6]. Inflammation is defined as physiological tissue damage or destruction resulting from diverse cytological and chemical responses that initiate a pathological state [7]. Physiological inflammation is an adaptive response to stress or physical exertion that exhibits a protective mechanism against stressful stimuli and facilitates tissue regeneration. However, chronic inflammation may evolve into a pathological condition, adversely impacting health and well-being, as well as an athlete’s career, resulting in diminished performance and weakened immune defense [8].

The innate immune system is the primary defensive mechanism of animals against many external threats, which facilitates a robust inflammatory response that offers immediate protection [7,9,10]. Under homeostatic conditions, leukocyte populations migrate between the vasculature and other tissues for immune surveillance. They are attracted to the site of inflammation in response to infections or injuries and play critical roles in pathogen clearance and tissue healing [11]. The adaptive immune response is modulated by T cells activated by antigen-presenting cells, specifically dendritic cells and macrophages. Effective interaction results in the rapid expansion and differentiation of naive T cells into effector CD4+ T lymphocytes. These cells release several cytokines that activate adaptive immune cells, such as macrophages and CD8+ T cells, to eradicate the infection. T lymphocytes can demonstrate regulatory functions by transmitting either positive (helper) or negative (suppressor) signals to other leukocytes or by exerting cytotoxic effects [12,13,14]. CD4+ T cells are essential for modulating autoimmune and allergic immune responses. CD8+ T cells are essential elements of the adaptive immune system that orchestrate cytotoxic responses [9]. The activity and reactivity of immune cells are reflected in the cytokines they secrete, such as interleukin IL-1α, IL-1β, IL-2, and IL-6, which are known to be potent anti- or pro-inflammatory cytokines [15,16]. Blood cells and cytokine production serve as biomarkers for health, illness, therapy response, and athletic performance monitoring in horses [17,18,19,20,21]. Therefore, it is necessary to consider that all biological systems, including immune and inflammatory responses, follow a temporal organization, where the magnitude and characteristics of their responses vary according to the phases of the biological clock [22,23]. Circadian clocks are present in immune cells and are essential for controlling the migration, production, and secretion of cytokines and inflammatory factors, as well as phagocytic activity, thereby influencing immunological and inflammatory responses in mammals [11,24,25,26,27,28,29,30,31,32,33]. The concentration of circulating B and T cells in the bloodstream peaks during daylight hours in mice, regulated by the gene expression of their chemokine receptors, influenced by their diurnal or nocturnal chronotype [34]. The intensity of the pulmonary response to endotoxin exposure is intensified during daylight hours in mice, influenced by neutrophil circadian rhythms [34]. Additionally, the timing of host infection affects the progression of viral disease in mice, and the presence of an operational circadian clock enhances the efficacy of the immune response [35]. Circadian clocks regulate leukocyte migration across the body, successfully controlling the quantity of leukocytes in designated areas during the day in humans [36]. Furthermore, leukocyte effector functions exhibit diurnal variation, as does the ability of activated immune cells to multiply and synthesize cytokines, which are likely developed to protect against daily peaks in pathogen exposure [11,12,13,14,15,16,17,18,19,20,21,22,23,24,25,26,27]. In diurnal species, such as horses, it has been demonstrated that the greatest concentration of immune cells occurs at night [37,38]. Similar implications have been observed in humans, where circulating T-cell counts exhibit diurnal fluctuations, peaking during the behavioral rest period and decreasing by up to 40% at the peak of the active phase [31]. Likewise, the quantity of circulating effector CD8+ T cells reaches its peak during the active phase, potentially enhancing immunological defense when damage or infection is most probable [39]. Recent research indicates that the circadian clock not only governs immunological responses but is also influenced by components of the immune system; hence, the interaction between the immune system and the circadian clock is bidirectional [22,38,40,41,42]. Consequently, disruption of the immunological circadian rhythm may result in immune system dysfunction, dysregulation of the inflammatory response, heightened vulnerability to infections and diseases, and diminished athletic performance, exacerbating inflammatory responses and economic losses in equine practice [11,25]. Considering the significance of biological rhythms in parameters observed in both humans and mice, it would be intriguing to investigate how the immune and inflammatory systems in horses are influenced by daily rhythms, considering white blood cell count (WBCs), leukocyte subpopulations, CD4+, CD8+ populations, interleukin-1β (IL-1β), interleukin-6 (IL-6), and tumor necrosis factor-α (TNF-α) in horses.

## 2. Materials and Methods

All animal-handling procedures were conducted according to the principles outlined in the Declaration of Helsinki, the directive 2010/63/EU of the European Parliament and of the Council of 22 September 2010 on the protection of animals used for scientific purposes, and approved by the Ethical Committee of the University of Messina (06/2023).

Ten Italian Saddle horses (five non-pregnant, non-lactating mares and five geldings), aged 7 to 12 years, with a mean body weight of 480 ± 30 kg, were enrolled in the experimental study with the owner’s informed consent. The horses’s physiological and clinical status were checked by monitoring heart rate, respiratory rate, rectal temperature, fecal consistency, and hematological and hematochemical profiles, and animals that had injuries or did not have physiological range values for horses were excluded from the study. The subjects included were free from internal and external parasites (regularly treated every three months), regularly subjected to Coggins tests (one time a year), and vaccinated against influenza and tetanus. The yearly vaccines were administered three months before the experimental period, with the last immunization deferred until the conclusion of the trial. The experimental group was maintained in identical individual boxes of 3.5 × 3.5 m and 6 m in height at a private horse training facility in Sicily, Italy (Latitude 38°7′ N; Longitude 13°22′ E). Throughout the experimental duration, temperature, hygrometric measurements, and ventilation were assessed utilizing a multiparameter probe (Testo 400). Each box was fitted with a 1.5 × 1.5 m window, and a grid was positioned in front of the box wall to facilitate social interaction. The horses were provided with a specialized diet for trained equines, consisting of high-quality hay and concentrates (crude protein 16%, crude fat 6%, crude fiber 7.35%, ash 10.09%, sodium 0.46%, lysine 0.85%, methionine 0.35%, and omega-3 0.65%). Water was accessible ad libitum, and the animals were typically ridden every other day in the afternoon. The horses were subjected to daily show-jumping training. During the experimental period, the weekly training program was suspended from the day before, and the horses were kept at rest.

### 2.1. Sampling

Blood samples were collected every 4 h for 24 h, starting from 13:00 during the weekly rest (sunrise 05:05, sunset 20:45). Sampling was performed using a catheter (FEP G14; 13.5 cm) inserted into the jugular vein and affixed with a suture and bandage. The animals were accustomed to handling and pharmacological treatments, similar to sport horses. Therefore, catheterization was performed to mitigate the stress of the animal possibly caused by the manipulation during the blood draw sustained every 4 h. The catheter was fitted with an extension tube to enable blood collection into vacuum tubes containing ethylenediaminetetraacetic acid (EDTA) and a clot activator to obtain serum. Immediately after blood collection, a blood smear was prepared from the EDTA tubes. All blood samples were moved to the laboratory, stored at refrigeration state (4 °C), and complete blood count was performed within 2 h. Subsequent to air-drying, the slides were stained using the May-Grünwald Giemsa mixture stain technique [1]. Leukocyte identification and quantification were performed on all samples using a manual 100-cell differential count to classify neutrophils, basophils, eosinophils, lymphocytes, and monocytes in each blood smear. White blood cells (WBC were evaluated using an automated hematology analyzer (HeCo Vet C; SEAC, Florence, Italy).

### 2.2. Flow Cytometry Analysis

Flow cytometry was performed on EDTA samples to determine the CD4+ and CD8+ cell subpopulations.

Initially, antibody standardization was performed to determine the minimum amount of antibody necessary to obtain the best MESF response (equivalent soluble fluorochrome molecules). Titration of the monoclonal antibody was performed to obtain the best result with the least amount of antibody (between 1:20 and 1:200). Monoclonal antibodies (Biorad) were used to determine the lymphocyte subpopulations, CD4 and CD8, conjugated with different fluorochromes (FITC and PE). Draq5 (nuclear dye) was used to stain the nuclei to provide better image contrast. The analysis was performed on the EDTA sample to obtain biparametric assays from a single blood sample. The antibodies are reactive against equine species [43,44,45]. The CD8 clone CVS8 and the CD4 clone CVS21. EDTA samples (100 µL) were transferred to empty tubes, and 3 µL of the respective markers were added. Samples in EDTA were initially incubated for 30 min in the dark to avoid fluorochrome degradation. The analytical method utilizing Image Stream does not require the lysis of red blood cells, as they are excluded through gating analysis of lymphocyte populations. However, following incubation with monoclonal antibodies, the cells were lysed using an ammonium chloride solution and subsequently fixed in a 0.5% paraformaldehyde solution to enhance the stability and performance of the samples.

Flow cytometric analysis was performed using a multispectral flow cytometer, ImageStreamX (Amnis, Seattle, WA, USA), in which standard microscopy was combined with flow cytometry. This instrument can acquire up to 100 cells/s, with the simultaneous acquisition of six images of each cell, including multiple fluorescent images, bright field, and scatter. In addition, the integrated software INSPIRE (version 201.1.0.765) runs on the ImageStreamX Mark II. Each prepared sample was maintained on ice before being run into the flow cell. The cells were then allowed to form a single-core stream before acquisition. Images were analyzed using the IDEAS image-analysis software (Amnis). Approximately 10,000 cells were acquired and analyzed to assess the percentage of CD4 and/or CD8-positive cells in the sample.

### 2.3. Identification of CD4+ and CD8+ Subpopulations

The analysis of the CD4+ and CD8+ lymphocyte subpopulations was performed within a pool of previously selected, focused, and isolated leukocyte cells.

**Figure 1:**
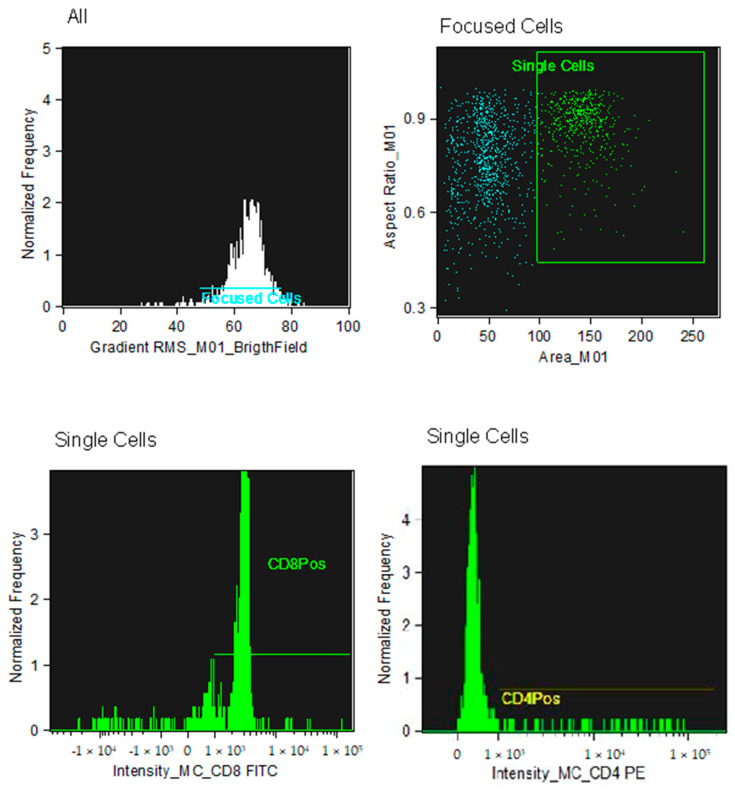
Scatter plots show the recruitment of focused lymphocytes and the area where the marked cells were found with antibodies. The images are representative and were chosen at random.

**Figure 2:**
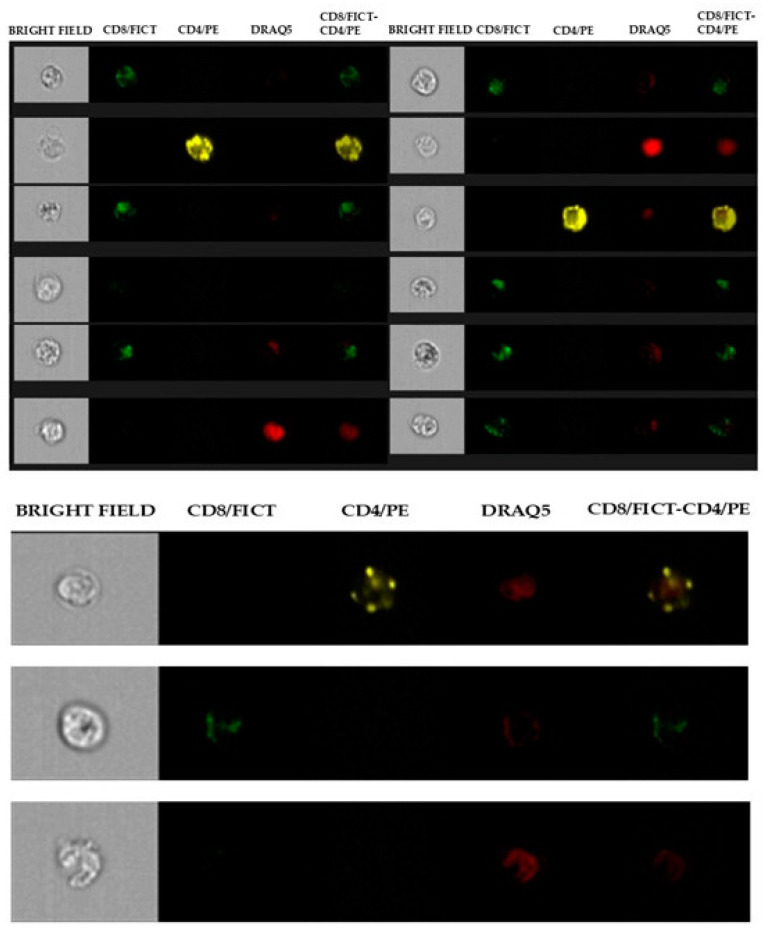
Single marked cells with CD8-Fict, CD4-PE nucleus-Draq5, and unmarked cells. The images are representative, chosen at random.

**Figure 3:**
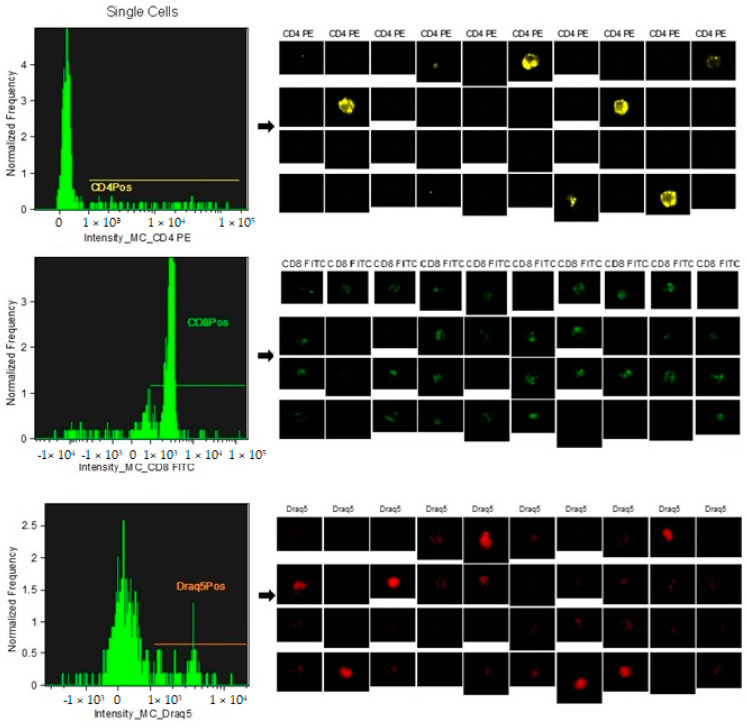
Fluorescent intensity graphs and images of compensated data. The cells were labeled with individual fluorochromes and imaged simultaneously (FITC, PE, and DRAQ5). After compensation, the graphs show a good representation of the labeled populations that are well compensated and without overlapping. The images are representative, chosen at random.

The analysis technique does not involve an actual gating strategy, like classical flow cytometry, but a selection of images (gradient) of the clear field of positively fluorochrome-marked lymphocyte cells that are in focus. For the images that are in focus, the size and appearance of individual cells are identified so that they can be shown at the graphical level as images. Within the selected areas, the cells superficially marked with CD4+ and those marked with CD8+ and the nucleus were highlighted to better distinguish the internal from the superficially marked components of the individual cells observed, as shown in Figure 1 and Figure 2. The lymphocyte identification strategy involves the use of the same two monoclonal antibodies that identify the T population of lymphocytes. The IDEAS^®^ Compensation Wizard was used to generate a compensation matrix using single-color fluorescent cells that were collected on the ImageStream in the absence of brightfield illumination and side scatter (SSC). One file of between 500 and 1000 positive events was collected for each fluorochrome in the experiment, using the instrument configuration according to the manufacturer’s start guide: Compensation 4.0, as shown in Figure 3. The cutoff between negative and positive is related to the high sensitivity of the experiment; therefore, there is no need to use an isotype control, which is required in classical flow cytometry. Unstained samples were evaluated as controls, as described in previous studies using the same technique in horses [45]. In horses, ideal isotype controls have not been identified to be able to define the binding specificity of the monoclonal antibody used (high purity) for imaging flow cytometry. Indeed, staining with the monoclonal antibody alone was used to determine non-specific signals.

### 2.4. Interleukine Analysis

On obtained serum samples, the concentrations of interleukins 1β, 6, and tumor necrosis factor-α (IL-1β, IL-6, and TNF-α) were assessed using enzyme-linked immunosorbent assay kits specific for equine species (Equine IL-1β ELISA kit, sensitivity 3.5 pg/mL; Intra-Assay CV% < 10%, and Inter-Assay CV% < 12%, determining reproducibility RayBio^®^, Peachtree Corners, GA, USA Equine TNF- α ELISA kit, sensitivity 0.64 pg/mL; Intra-Assay CV% < 10%, and Inter-Assay CV% < 12%, determining reproducibility RayBio^®^; Equine IL-6 ELISA kit, sensitivity 5.5 pg/mL; Intra-Assay CV% < 10%, and Inter-Assay CV% < 12%, determining reproducibility RayBio^®^) using a micro-well plate reader (Sirio, SEAC, Florence, Italy). This study did not include intracellular cytokine staining as a limitation. All calibrators and samples were run in duplicate, and the samples exhibited parallel displacement to the standard curve for each ELISA analysis. The concentrations of IL1β, IL-6, and TNFα were obtained from the resulting optical densities (OD) used to calculate each standard curve.

### 2.5. Statistical Analysis

Data were normally distributed (Kolmogorov-Smirnov test, *p* > 0.05) and are reported as mean ± standard deviation (SD). The periodic phenomenon was analytically evaluated by the application of a trigonometric statistical model on each obtained value at each time point to assess the main rhythmic parameters: MESOR, amplitude (the difference between the peak, or trough, and the mean of a wave), acrophase (when the peak of a rhythm occurs), and robustness (strength of rhythmicity) using the single COSINOR procedure. Whereas MESOR and amplitude of different rhythms cannot be compared because they refer to distinct physical quantities, the analysis of the temporal relationship of physiological processes considers the comparison of acrophase and robustness of rhythm. A one-way analysis of variance (ANOVA) was applied to identify the differences in acrophase and robustness among all the investigated parameters. Bonferroni post hoc test was performed to observe multiple comparisons. A multiple correlation analysis model (Pearson) was computed to evaluate the relationship among the investigated parameters. Differences were considered statistically significant at *p* < 0.05. The 95% confidence intervals for the data recorded under the experimental conditions were determined. Data were analyzed using the Prism v. 9.4.0 (Graphpad Software Ltd., Solana Beach, CA, USA).

## 3. Results

**Figure 4:**
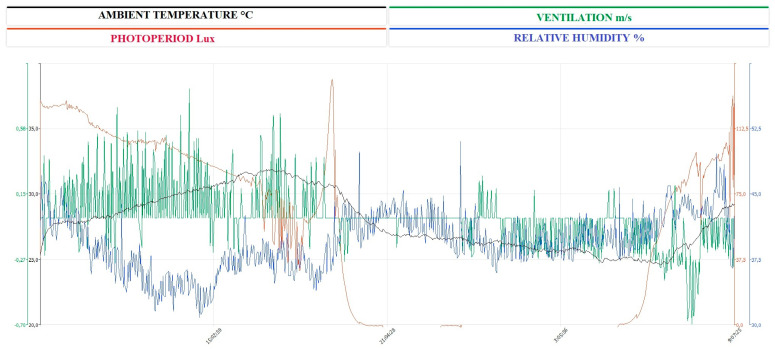
Environmental trends of the analyzed parameters during the 24 h. Black lines represent ambient temperature (°C), green lines represent ventilation (m/s), red lines represent photoperiod (lux), and blue lines represent relative humidity (%) measured using a Testo-400.

**Figure 5:**
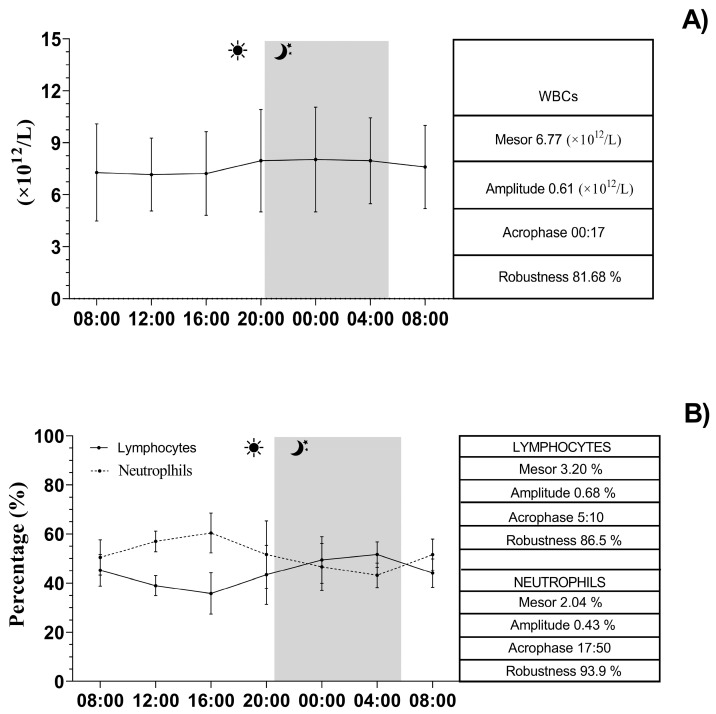
Representative records of WBCs (**A**) and lymphocytes and neutrophils (**B**) rhythm in horses. The gray bar indicates the start of the dark phase of the light-dark cycle during the 24 h. COSINOR analysis is described in the boxes on the right.

**Figure 6:**
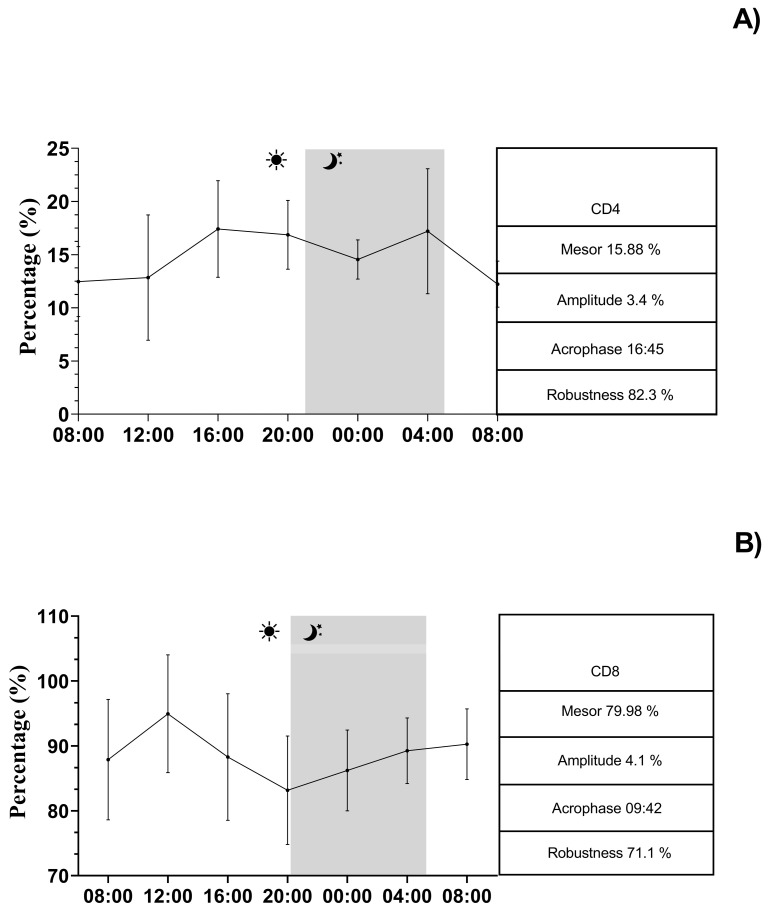
Representative records of CD4+ (**A**) and CD8+ (**B**) rhythms in horses. The gray bar indicates the start of the dark phase of the light-dark cycle during the 24 h. COSINOR analysis is described in the boxes on the right.

**Figure 7:**
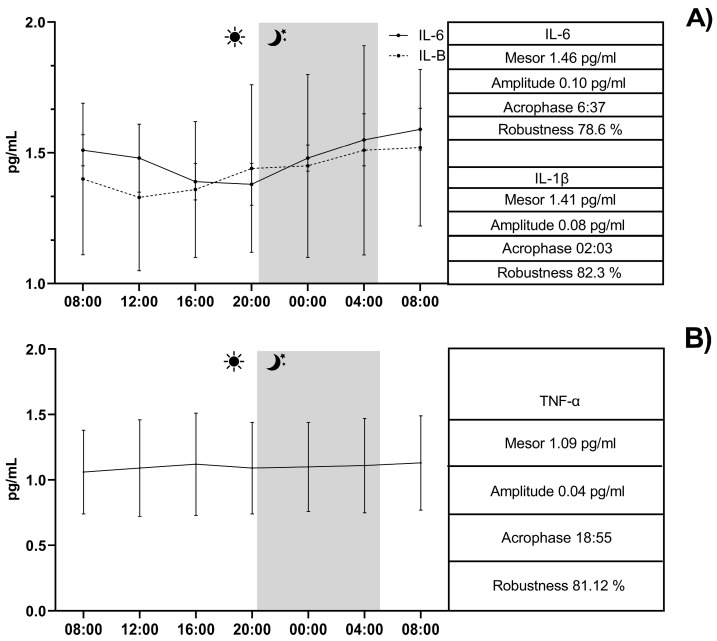
Representative records of IL-6 and IL-1β (**A**) rhythm and TNFα (**B**) in horses. The gray bar indicates the start of the dark phase of the light-dark cycle during the 24 h. COSINOR analysis is described in the boxes on the right.

**Figure 8:**
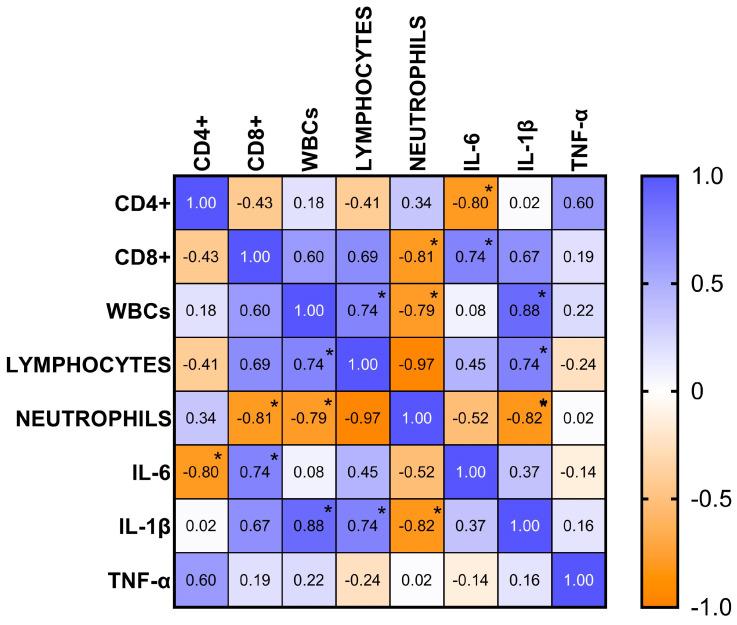
Heat map of multiple correlation analysis (r-values) of CD4+, CD8+, WBCs, lymphocytes, neutrophils, IL-6, and IL-1β. The symbol * represents *p* < 0.01.

The continuous recording of the ambient temperature (°C), relative humidity (%), ventilation (m/s), and photoperiod (lux) over a 24-h period conducted inside the boxes is shown in Figure 4. The application of the single COSINOR procedure showed a daily rhythmicity of WBCs, neutrophils, lymphocytes, CD4+, CD8+, IL-1β, IL-6, and TNF-α. The daily rhythms observed for each parameter are shown in Figure 5, Figure 6 and Figure 7. In particular, WBCs and IL-1β showed a nocturnal acrophase, IL-6 and lymphocytes peaked during the early morning hours, and neutrophils, CD4+, CD8+, and TNF-α showed a diurnal acrophase. All parameters showed a robustness of rhythm higher than 70%. This parameter represents the magnitude of reproducibility (or the degree of “stationarity” of the time series) [46]. One-way ANOVA showed a statistical difference in the acrophase among the investigated parameters (*p* < 0.0001), as shown by the Bonferroni post hoc comparison in Table 1. No significant difference in robustness was observed (*p* = 0.72). The Pearson correlation matrix obtained for each parameter is shown in Figure 8.

## 4. Discussion

Many physiological activities in horses demonstrate a daily cycle. The immune system provides “reactive” responses to changes in physiological variables for antigenic challenge to restore homeostasis, which is regulated at the circadian level (anticipating environmental cues before they occur) [47,48,49,50]. The examined physiological parameters, including WBCs, CD4+, CD8+, lymphocytes, neutrophils, and interleukins IL-6, IL-1β, and TNFα, demonstrated daily rhythmicity with precise temporal positioning, aligned with their distinct activities in the body. Specifically, white blood cells and lymphocytes demonstrated a daily rhythm with an acrophase occurring at night, similar to observations in humans [31,39,51]. An opposing trend has been noted in mice during the rest period (daytime), and given its nocturnal behavior, it may be confirmed that a diurnal animal like the horse, exhibits a nocturnal rhythm of white blood cells and lymphocytes [52,53].

A daily rhythmic expression of clock genes (Bmal1, Cry 1, Per 1, Per 2, and Per 3) has been observed in the peripheral lymphocytesin horses [54]. It was found that certain leukocyte subpopulations, such as polymorphonuclear neutrophils, were predominant during an endotoxic event and responded to the inflammatory mediator prostaglandin E2 through the upregulation of certain clock genes (Per2 and Bmal1). These results provide the first evidence for the potential role of clock genes in the function of certain leukocyte subpopulations during the innate immune response in horses. More recently, stimulation with lipopolysaccharide of whole blood collected from horses at 4-h intervals over a 24-h period indicated that the response of cytokines and clock genes to an antigenic challenge varied significantly throughout the day. In particular, interleukin-6, an important immunomodulatory cytokine involved in the differentiation of Th1/Th2 immune responses [41], showed greater upregulation during the evening hours. This finding strongly suggests that there may be an optimal time of day for vaccine administration in horses to ensure maximum immunization and subsequent protection [23]. Nevertheless, findings on CD4/CD8 oscillations in other published flow cytometry-based studies in horses are absent. Daily fluctuations in blood leukocyte counts have been ascribed to a rhythmic redistribution of cells between peripheral tissues and circulating blood compartments, as well as to the rhythmic production of new cells, as already observed in horses [38]. Similar to other results obtained in horses and humans, lymphocytes peaked during the early morning hours and neutrophils during the afternoon, with a high percentage of rhythm [34,38]. This rhythmic distribution displays the physiology of the immune system during the horse’s active phase, when antigen entry is most likely, necessitating significant energy expenditure [54]. In the early morning, the autonomic nervous and neuroendocrine systems have been demonstrated to influence leukocyte physiology, reinforcing the notion that circadian timing is a crucial element of hypothalamic–immune communication in humans [55]. Furthermore, neutrophils, CD4+, and CD8+ (T suppressor/cytotoxic) cells exhibit peak levels throughout the day, likely to facilitate a temporal organization of cellular immune function [34,35,36,37,38,39,40,41,42,43,44,45,46,47,48,49,50,51]. Neutrophil populations, as known in humans, interact with dendritic cells and macrophages, presumably modulating T cell activation [56,57]. They were recruited during infections mediated by the expression of the core clock gene Arntl (encoding Bmal1), entrained by light, and regulated by glucocorticoids in a circadian manner [58,59]. Distinct T-cell subsets have differential migratory patterns throughout the day, governed by fluctuations in serum levels of glucocorticoids and catecholamines [11]. CD4+ and CD8+ T cells represent two entirely separate immune cell compartments characterized by markedly divergent temporal dynamics and activation criteria. One of the key cells involved in the adaptive immune response is CD8+ T cells, which eradicate intracellular infections, manage persistent infections, and tumors by generating cytotoxic chemicals (granzyme and perforin) and cytokines (IL-2, IFN-γ, and TNF-α) in humans [33]. The cytotoxic function of CD8+ T cells in infectious disorders has been documented in humans, with levels diminished at night and peaking around midday [46], while mice display the opposite trend [33]. This behavior may facilitate the body’s preparation for the demands of active periods to combat pathogens, whereas elevated nocturnal levels of CD4+ cells may be associated with their regulatory function. Consequently, the varying behavior of lymphocyte subpopulations may account for temporal alterations in the intensity and/or manifestation of immunological responses, such as fighting infection and disease [51]. The trafficking of leukocyte cells in both healthy and pathological settings is governed by rhythmic cytokine production and/or temporal expression of adhesion molecules on the cell surface [28]. Biological rhythms are crucial for immunological homeostasis and control the diurnal rhythmicity of leukocyte trafficking in both inflammatory and steady-state settings. Clock gene expression can be inhibited by inflammation, indicating a reciprocal and intricate link between the two biological systems [11,23,54]. The current investigation found a rhythmic oscillation of cytokines, as previously shown in humans and mice [8]. Specifically, IL-1β peaks at night, while IL-6 has a daily rhythm with its acrophase occurring in the early morning hours, as reported in humans [60,61,62,63,64]. IL-6 is one of the major cytokines that stimulates the hypothalamic−pituitary−adrenal (HPA) axis during inflammatory stress [65]. The peaks in circulating lymphocytes and cytokine production were synchronous, raising the possibility that diurnal variation in cytokine production is a consequence of variations in circulating cell numbers. TNF-α demonstrates rhythmicity, with an acrophase occurring during daylight hours, especially in the afternoon. TNFα is a pleiotropic cytokine that plays a pivotal role in the regulation of apoptosis, inflammation, and immunity. Its expression showed regional differences in the brain of rats, and its expression was at the highest level in the hypothalamus, hippocampus, and cerebral cortex at the beginning of the light phase [37,66].

This knowledge may be elucidated by the understanding that this parameter is involved in sleep regulation in humans and animals [61]. It has been noted that it is intricately linked to melatonin synthesis in both mice and humans, regarded as the primary synchronizer for the sleep-wake cycle [37]. TNF-α consequently induces nocturnal melatonin release, which subsequently reduces TNF-α levels in the bloodstream during the night in diurnal species like horses [67]. Based on the statistical results obtained through the analysis of acrophases (Table 1), they reflect the results of the rhythms and functions of the individual parameters analyzed, which are organized in the organism at different timings. In addition, it has also been observed that these parameters are positively and negatively correlated with each other. Based on the present results, the correlations strengthen the obtained findings. Specifically, IL-6 was negatively correlated with CD4+ production and positively correlated with CD8+; neutrophils were negatively correlated with CD8+, WBCs, and IL-1β. Conversely lymphocytes were positively correlated with WBCs and IL-1β, and WBCs were positively correlated with IL-1β, reflecting the functions and relationships of these parameters, as previously described. According to the peaks of the rhythms of the analyzed parameters, it can be observed that when IL-6 increased, whose peak was observed at 06:37, the value of CD8+, which actually peaks at 09:42, increased significantly, and the concentration of CD4+, which peaked in the afternoon hours, decreased significantly. Similarly, the high neutrophil concentration at 17:50 was negatively correlated with CD8+, WBCs, and IL-1β, which had a lower concentration at that time. Lymphocytes were also found to have the highest concentration during the night hours, similar to WBCs and IL-1β, which also had a night peak. It is widely known that the trends of the analyzed parameters, based on their immune and inflammatory functions, are influenced by exogenous factors such as physical exercise, illness, or stress [68,69]. However, no studies have investigated how these factors may concurrently influence the physiological rhythm of these parameters. The study of the biological rhythms of the immune and inflammatory parameters is essential not only to gain a better understanding of the physiology of these parameters in the animal kingdom, but more importantly, it is useful to organize the optimal time of day for drug administration and vaccination to evaluate a more efficient immune response by the organism. It would also be useful to determine the optimal time of day for physical training, considering the daily oscillation of inflammatory and immune responses in horses. The present research included a powerful and novel investigative tool, imaging flow cytometry, to investigate the concentration of certain lymphocyte subpopulations involved in immune system function [44,45]. These data demonstrate the reliability of imaging flow cytometry based on what has already been observed in humans and represent the beginning of an innovative approach in equine research, where such studies have previously been conducted using traditional flow cytometry [43,70].

## 5. Conclusions

The present study showed that the parameters studied, such as WBC, CD4+, CD8+, IL-1β, IL-6, and TNFα, display a daily rhythm in horses. This result represents a preliminary contribution to the knowledge of the influence of the daily rhythm physiology of the immune and inflammatory responses in horses. Therefore, the study of the daily rhythm of some immune and inflammatory components is essential for animal management and athletic performance of horses. This is crucial for the development of good management or training regimes for equine athletes and disease prevention, considering a potential risk window for infection and inflammation to which the horse may be susceptible.

## Figures and Tables

**Table 1:** Statistical differences in the results of acrophase observed among all the investigated parameters. The filled boxes show significant *p*-values, and the empty boxes show no significant differences.

Acrophase	CD4 +	CD8 +	WBCs	Lymphocytes	Neutrophils	IL-6	IL-1β	TNF-α
CD4+		<0.0001	<0.0001		<0.001	<0.0001	<0.0001	
CD8+	<0.0001		<0.01	<0.0001				<0.0001
WBCs	<0.0001	<0.01		<0.0001	<0.001			<0.0001
Lymphocytes		<0.0001	<0.0001		<0.0001	<0.0001	<0.0001	
Neutrophils	<0.001		<0.001	<0.0001			<0.01	
IL-6	<0.0001			<0.0001				<0.0001
IL-1β	<0.0001			<0.0001	<0.0001			<0.0001
TNF-α		<0.0001	<0.0001		<0.0001	<0.0001	<0.0001	

## Data Availability

The raw data supporting the conclusions of this article will be available from the authors upon request.
